# Enhancing Decision-Making in Pediatric Radiotherapy: Internal Validation and Expansion of the M.A.P.-RT Schedule for Sedation Need Prediction and Identification of Psychological Support Needs in a Prospective Cohort of 268 Pediatric Patients

**DOI:** 10.3390/children13101328

**Published:** 2026-09-30

**Authors:** Elisa Marconi, Elisa Meldolesi, Silvia Mariani, Marika Mauti, Nicola Dinapoli, Mariangela Massaccesi, Loredana Dinapoli, Lucrezia Bernabucci, Sara Iacovone, Gabriele Turco, Antonella Guido, Daria De Cristofaro, Andrea De Salvo, Giulia Panza, Gerardina Stimato, Antonio Ruggiero, Angela Mastronuzzi, Giulia Zucchetti, Patrizia Cornacchione, Vincenzo Frascino, Luca Tagliaferri, Daniela Pia Rosaria Chieffo, Maria Antonietta Gambacorta, Silvia Chiesa

**Affiliations:** 1 Clinical Psychology Unit, Fondazione Policlinico Universitario Agostino Gemelli IRCCS, 00168 Rome, Italy; elisa.marconi@policlinicogemelli.it (E.M.); marika.mauti@guest.policlinicogemelli.it (M.M.); loredana.dinapoli@policlinicogemelli.it (L.D.); antonella.guido@guest.policlinicogemelli.it (A.G.); danielapiarosaria.chieffo@policlinicogemelli.it (D.P.R.C.); 2 Radiation Oncology Unit, Department of Diagnostic Imaging and Oncological Radiotherapy, Fondazione Policlinico Universitario Agostino Gemelli IRCCS, 00168 Rome, Italy; elisa.meldolesi@policlinicogemelli.it (E.M.); nicola.dinapoli@policlinicogemelli.it (N.D.); mariangela.massaccesi@policlinicogemelli.it (M.M.); lucrezia.bernabucci@guest.policlinicogemelli.it (L.B.); sara.iacovone@guest.policlinicogemelli.it (S.I.); gabriele.turco@guest.policlinicogemelli.it (G.T.); giulia.panza@policlinicogemelli.it (G.P.); vincenzo.frascino@policlinicogemelli.it (V.F.); luca.tagliaferri@policlinicogemelli.it (L.T.); mariaantonietta.gambacorta@policlinicogemelli.it (M.A.G.); silvia.chiesa@policlinicogemelli.it (S.C.); 3 Department of Psychology, Università Cattolica del Sacro Cuore, 20123 Milano, Italy; daria.decristofaro01@icatt.it; 4 Pediatric Hematology and Oncology and Cell and Gene Therapy, IRCCS Bambino Gesù Children’s Hospital, 00165 Rome, Italy; a.desalvo@fastwebnet.it (A.D.S.); angela.mastronuzzi@opbg.net (A.M.); 5 Medical Physics Unit, Department of Diagnostic Imaging and Oncological Radiotherapy, Fondazione Policlinico Universitario Agostino Gemelli IRCCS, 00168 Rome, Italy; gerardina.stimato@policlinicogemelli.it; 6 Pediatric Oncology Unit, Fondazione Policlinico Universitario Agostino Gemelli IRCCS, 00168 Rome, Italy; antonio.ruggiero@policlinicogemelli.it; 7 Pediatric Hematology and Oncology Unit, AO Regina Margherita–Sant’Anna, 10126 Turin, Italy; giulia.zucchetti@unito.it; 8 Department of Public Health and Pediatric Sciences, University of Turin, 10124 Turin, Italy; 9 Department of Radiological and Hematological Sciences, Università Cattolica del Sacro Cuore, 00168 Rome, Italy; patrizia.cornacchione@policlinicogemelli.it; 10 Department of Life Sciences and Public Health, Università Cattolica del Sacro Cuore, 00168 Rome, Italy

**Keywords:** paediatric radiotherapy, procedural sedation, psychosocial screening, predictive model, internal validation, supportive care

## Abstract

**Highlights:**

**What are the main findings?**

- In a prospective cohort of 268 children and adolescents, the M.A.P.-RT schedule predicted the need for procedural sedation during radiotherapy with excellent discrimination, confirming the performance reported in the RADAR pilot study.
- The same instrument discriminated the intensity of psychological support far less well, consistent with the findings of the previous study. The domains driving the two outcomes differed: developmental and medical-collaboration items predicted sedation, whereas treatment-burden items (minutes required per fraction, physical skills for RT) were stronger predictors of the need for intensive psychological support.
- The structured collection of psychosocial and clinical information also enabled further analyses of the psychological aspects of pediatric radiotherapy, with important implications for individualized care planning and treatment personalization. Age, not gender, was the principal demographic determinant of psychosocial vulnerability. Compared with adolescents and young adults, children aged 11 years or younger showed greater psychological difficulties and behavioural reactions on first entering the treatment room.

**What is the implication of the main finding?**

- A structured multidimensional assessment completed at the first radiotherapy consultation can identify, before treatment starts, which children are likely to require sedation, supporting targeted procedural preparation and a reduction in avoidable anesthesia exposure.
- Because the psychological-support outcome is predicted only modestly, the M.A.P.-RT could be used not only to trigger a dedicated psychological assessment and support the allocation of psychosocial resources, but also to personalize the delivery of psychological interventions and to provide a baseline for future studies evaluating the effectiveness of psychosocial care.

**Abstract:**

**Background:** Pediatric radiotherapy is often associated with high levels of psychological distress and vulnerability, affecting both patients and their families. In younger children, sedation is frequently required to ensure safety and adequate immobilization during treatment. The original RADAR Project demonstrated that the Multidimensional Assessment for Pediatric Patients in Radiotherapy (M.A.P.-RT) tool accurately predicts the need for sedation in pediatric patients undergoing radiotherapy. The present study aims to extend the validation of the M.A.P.-RT, including a larger cohort of patients, to confirm the tool’s predictive robustness and to identify clinical and psychosocial factors associated with sedation requirements, and to profile psychosocial and behavioural vulnerability according to age and gender. **Methods:** Pediatric patients referred for RT were prospectively enrolled and assessed with the M.A.P.-RT schedule during the first multidisciplinary consultation. Two outcomes were analysed: the need for procedural sedation and the level of psychological support required (intensive vs. standard). The questionnaire items, domain scores, and total score were examined to identify the factors most strongly associated with these outcomes. Predictive models were then developed using the domain scores, while the total score was evaluated separately. Model performance was assessed by repeated, fully nested 10 × 10-fold cross-validation (internal validation only), and odds ratios with 95% confidence intervals were calculated to estimate the strength of the associations. Linear regression models with age group (children ≤ 11 years vs. adolescents and young adults ≥ 12 years), gender, and their interaction were then fitted to test demographic predictors of psychological difficulties and behavioural reactions at treatment entry. **Results:** A total of 268 patients (mean age 9.6 years, SD 5.7; 59.3% male) were included in the analysis. Procedural sedation was required in 22.8% of patients, while 51.1% received intensive psychological support. The M.A.P.-RT showed excellent ability to identify children requiring sedation, with previous collaboration during radiotherapy procedures, age-related discomfort, and higher questionnaire scores emerging as the main predictors. The overall questionnaire score alone also provided high predictive accuracy. In contrast, the tool was less effective in distinguishing patients who required intensive psychological support: it misclassified roughly one patient in three, with a significant bias toward over-classifying patients into the higher-need category. Age group, but not gender, predicted psychosocial vulnerability: compared with adolescents and young adults, children aged ≤11 years showed significantly greater psychological difficulties, parental anxiety, post-diagnosis distress, and externalising coping and patient-management difficulties and more pronounced reactions across all five dimensions of the Children’s Emotional Manifestation Scale (CEMS), included in the M.A.P.-RT, at treatment entry. **Conclusions:** In this large single-centre cohort, and under internal validation only, the M.A.P.-RT confirmed its value as a reliable tool for predicting the need for procedural sedation in pediatric radiotherapy, while its ability to predict the intensity of psychological support was more limited, indicating that it is better used to prompt psychological assessment than to allocate support; this was probably due to different properties of the two targets. Age was the main profiling variable, identifying younger children as systematically more vulnerable; sex was not associated with any outcome. These findings highlight that integrating structured psychosocial information into patient profiling may facilitate the early identification of individual vulnerabilities and support the delivery of more personalized psychosocial care. They also underscore the potential of multidisciplinary assessment tools to enable the early identification of individual needs and vulnerabilities, supporting the development of personalized supportive interventions to improve the radiotherapy experience of children and their families. Because validation was internal and conducted in a single centre, external validation remains necessary before the schedule is adopted elsewhere.

## 1. Introduction

In pediatric oncology, radiotherapy is required by roughly one-third of children treated for cancer, and its safe delivery depends on a degree of cooperation that many young patients cannot sustain unaided [1]. Accurate dose delivery requires an individualised immobilisation system and complete stillness, first during simulation and then throughout every fraction [2]. When a child cannot maintain a reproducible position, sedation or general anesthesia becomes necessary.

Anesthesia solves the immobilisation problem but introduces others. Daily sedation disrupts the child’s routine and requires prolonged fasting; it demands the presence of an anesthesiology team for the entire treatment slot, lengthens room occupancy and raises the cost of each course [3]. A recent systematic review by the SIOP Europe Working Group on Pediatric Anesthesia in Radiation Therapy documented that, despite the widespread use of sedation and general anesthesia in this setting, standardised protocols regarding agents, staffing and monitoring are still lacking across institutions, and highlighted concerns about acute complications and potential long-term neurodevelopmental effects of repeated exposure [4]. Age emerged in that review as one of the most consistent determinants of need, with sedation almost universal in the youngest children and progressively rare through late childhood—an observation mirrored by single-institution series reporting general anesthesia in over 90% of children under three years of age against well under 5% in adolescents [5].

These considerations have motivated a substantial body of work on non-pharmacological alternatives. Play-based procedural preparation, structured psychoeducational programmes, the involvement of dedicated child life specialists, and audiovisual distraction during treatment have all been shown to reduce anesthesia use, sometimes markedly [6,7,8,9]. In a prospective multicentre trial conducted across ten institutions, video-based distraction delivered through the AVATAR system allowed 78% of children aged 3–10 years to complete radiotherapy without anesthesia, a rate significantly higher than in historical comparison series [9]. Immersive virtual reality and purpose-built serious games have similarly been explored as preparatory tools, with encouraging feasibility and acceptability data [10,11]. Qualitative work in children undergoing proton therapy indicates that those who remain awake describe improving self-efficacy across the treatment course and identify structured preparatory materials as helpful in reducing pre-treatment fear [11].

What these interventions share is a dependence on knowing, in advance, which children will benefit. Preparation programmes are staff-intensive and cannot be delivered at maximal intensity to every patient; conversely, defaulting to sedation for all young children exposes many to an intervention they would not have required.

The broader psycho-oncology literature has increasingly framed systematic assessment as the entry point to a matched, tiered model of care, in which the intensity of support is calibrated to each patient’s estimated level of risk rather than distributed uniformly. The Pediatric Psychosocial Preventative Health Model (PPPHM) [12] formalised this approach, and the 2015 Standards for the Psychosocial Care of Children with Cancer and Their Families [13] recommend using psychosocial assessment to allocate limited resources according to need.

Building on the Pediatric Psychosocial Preventative Health Model (PPPHM), the Psychosocial Assessment Tool (PAT) was specifically developed as its clinical operationalization, translating the model’s risk stratification framework into a standardized screening instrument for identifying family psychosocial risk and guiding the allocation of psychosocial care. In pediatric oncology more broadly, systematic psychosocial screening is an established standard of care, and the Psychosocial Assessment Tool (PAT) is the most widely implemented instrument for that purpose [14,15]. Risk classification obtained with the PAT at diagnosis predicts subsequent psychosocial service utilisation and family distress across treatment [16], and implementation studies across multiple cancer programmes have shown that universal screening is feasible when organisational barriers are addressed [17]. The PAT was not, however, designed for the specific procedural demands of radiotherapy: it does not capture the child’s tolerance of immobilisation, behaviour on first entering the treatment bunker, or the practical time burden of a fraction. The missing element is a structured, radiotherapy-specific instrument that stratifies need at the first clinical contact.

Consistent with evidence showing that developmentally tailored and family-centred preparation is more effective than generic approaches, the Multidimensional Assessment for Pediatric Patients in Radiotherapy (M.A.P.-RT) was developed to operationalise this principle within pediatric radiotherapy. By integrating psychosocial, developmental, clinical, and procedural information collected by the multidisciplinary team during the initial evaluation [18], it provides a structured basis for identifying children at higher risk of requiring procedural sedation and for directing preparatory and psychological resources where they are most likely to improve treatment adaptation.

In the original RADAR pilot study, the M.A.P.-RT schedule showed excellent discrimination for the need for procedural sedation and good discrimination for the need for intensive psychological support [18]. Those results, while promising, were derived from a modest sample, and the correspondingly wide confidence intervals left the stability of the individual predictors uncertain.

The present study was therefore designed to validate and expand the M.A.P.-RT in a substantially larger prospective cohort. The primary objective was to confirm the predictive performance of the schedule for procedural sedation. Secondary objectives were to re-examine its performance for the intensity of psychological support, to compare the discriminative ability of the composite Total Score against a multivariable model built on the six domain scores, and to identify which dimensions of the assessment carry independent predictive weight once the sample is large enough to estimate them with precision, as well as to identify other clinical and psychosocial predictors associated with the need for sedation or psychological support. In addition, we explored whether specific demographic profiles, particularly age- and sex-related characteristics, were associated with distinct patterns of psychological distress and behavioural responses at treatment entry. Identifying these profiles may facilitate risk stratification beyond the prediction of sedation alone, allowing clinicians to anticipate the emotional and behavioural needs of different patient subgroups and to tailor preparatory interventions accordingly. Such profiling is increasingly recognised as a key component of personalized pediatric oncology care, where matching the intensity and type of support to the individual child has the potential to improve cooperation during treatment, reduce unnecessary sedation, minimize treatment-related distress, and ultimately enhance both treatment precision and the overall radiotherapy experience for children and their families.

## 2. Materials and Methods

### 2.1. Procedure

This prospective observational study was designed to validate and extend the original M.A.P.-RT predictive model developed by Chiesa et al. [18]. Consecutive pediatric patients referred for radiotherapy were enrolled at the participating radiation oncology center during the study period (2019–2025). At the patient’s first visit, the radiation oncologist, nurse, and psychologist completed the M.A.P.-RT form (described in detail below and in Chiesa et al. [18]), a multidimensional assessment tool designed to capture the emotional, psychological, and relational characteristics of pediatric patients and their families.

### 2.2. Participants

This prospective observational study expanded and validated the M.A.P.-RT predictive model originally developed by Chiesa and colleagues [18] using a larger cohort of consecutive pediatric patients treated at a single radiation oncology centre.

Eligibility criteria were: a diagnosis of pediatric oncological or haematological malignancy requiring radiotherapy; radiotherapy delivered with curative, disease-control or palliative intent; treatment administered with or without procedural sedation; and availability of a complete M.A.P.-RT assessment performed before the start of treatment.

Two hundred and seventy patients were enrolled. Two patients aged 29 and 33 years were excluded as age outliers in order to preserve a strictly pediatric and young-adult range, yielding a final analytic sample of 268 patients aged 1–23 years. The upper boundary of 23 years was not a design cut-off but reflects the case-mix of our paediatric radiotherapy pathway. The original RADAR protocol enrolled patients aged 0–18 years together with patients aged 18–21 years with cognitive impairment; moreover, in our institution, adolescents and young adults with a paediatric-type malignancy, or whose treatment began in childhood, continue to be managed within the paediatric multidisciplinary programme and are assessed with the M.A.P.-RT at their first consultation. All such consecutively assessed patients were therefore retained, which set the observed upper limit at 23 years. The two patients aged 29 and 33 years fell outside the upper bound of any recognised adolescent and young adult definition and outside the developmental span the schedule was designed to cover, and were excluded on that basis rather than on statistical grounds.

Within the analytic sample, 169 patients were classified as children (≤11 years) and 99 as adolescents and young adults (AYA; ≥12 years) for the demographic analyses described below.

Written informed consent was obtained from all participants and/or their legal guardians prior to data collection. The study was conducted in accordance with the Declaration of Helsinki and approved by the Ethics Committee of Fondazione Policlinico Universitario Agostino Gemelli IRCCS, Rome (RADAR ID 3277; last amendment approved in May 2023).

#### 2.2.1. The M.A.P.-RT Schedule

The M.A.P.-RT schedule was administered during the first multidisciplinary radiotherapy consultation according to the original methodology [18]. It is completed jointly by the radiation oncologist, the nurse, the radiation therapy technician and the clinical psychologist, and yields numerical scores across six domains:

- A—Pain/distress, nursing observation. The nurse who first receives the child rates pain and distress using the observer-rated Wong–Baker scale (0–10).
- B—Development, discomfort-by-age score. Captures the expected impact of developmental stage on tolerance of the procedure, weighted for children between 0 and 10 years (0–10).
- C—Medical, first medical evaluation. The radiation oncologist records the information held by the family and the child the child’s collaboration during previous diagnostic imaging and with requests from parents and staff, tolerance of separation from the caregiver, and distress observed during the visit (0–22).
- D—Physical, skills for RT. Motor, cognitive and language difficulties relevant to positioning, together with the time required for the planned fraction (0–26).
- E—Emotional distress, first entry to the LINAC room. Behavioural and emotional reactions on first entering the treatment bunker, scored by the technician with the Children’s Emotional Manifestation Scale, CEMS (5–25).
- F—Psychological, clinical interview. Psychological dimensions judged to influence compliance during preparation and treatment, including past or current mental health problems, previous traumatic events, internalising and externalising coping styles, parental anxiety, and parenting and family difficulties (0–30).

Individual item scores, the six domain scores (Score A to Score F) and the overall Total Score were computed for each participant according to the original scoring system.

#### 2.2.2. Outcomes

Two binary outcomes were defined a priori.

Sedation was defined as the need for procedural sedation or general anaesthesia at any point during the radiotherapy course (yes/no). The two were treated as a single composite outcome because both require the presence of the anaesthesiology team, pre-procedural fasting and dedicated room time, and because the choice between deep procedural sedation and general anaesthesia is made by the anesthesiologist on pharmacological and airway-management grounds rather than on the basis of the child’s ability to cooperate (which is what the M.A.P.-RT measures). For consistency, the term “sedation” is used throughout the remainder of the manuscript to denote this composite outcome, and the terms “general anaesthesia” and “anaesthesia” are used only where the distinction is material.

Psychological support intensity was classified as intensive or standard on the basis of the ratio between the number of psychological sessions delivered and the number of radiotherapy fractions [18]. Applying the rule established in the pilot study, support was classified as intensive when this ratio exceeded 1/3, that is, when the patient received more than one psychological session for every three radiotherapy fractions (approximately more than one session every three treatment days), and as standard otherwise. The threshold was derived empirically in the original RADAR cohort as the median sessions-to-fractions ratio observed there and has not been validated against an independent clinical criterion.

#### 2.2.3. Relationship Between the Index Assessment and Outcome Ascertainment

The M.A.P.-RT is completed at the first consultation by the radiation oncologist, nurse, radiation therapy technician and clinical psychologist, independently rather than collegially. The multidisciplinary team is an extended team, with professionals not necessarily overlapping across different stages of the patient pathway. The M.A.P.-RT did not determine procedural sedation or psychological support, and no score threshold or prospective decision rule was applied. Anaesthesia was activated independently according to the established treatment-as-usual (TAU) clinical pathway, with the anaesthesiology team blinded to the M.A.P.-RT score; similarly, psychological sessions could be increased during radiotherapy according to emerging clinical needs, independently of the initial M.A.P.-RT assessment. Clinicians making the final assessment regarding sedation were therefore effectively blinded to the scores assigned by other professionals involved in the initial assessment. However, complete independence between index assessment and outcome ascertainment cannot be assumed, as some M.A.P.-RT indicators, such as cooperation during previous imaging, tolerance of caregiver separation and behaviour on entering the treatment room, may also inform clinical judgement regarding sedation and psychological support. Nevertheless, these observations were collected by independent assessors at different stages of the patient pathway, and the distributed structure of the multidisciplinary workflow substantially reduced the likelihood that the initial M.A.P.-RT assessment systematically influenced subsequent clinical decisions. Incorporation bias and, for sedation, an element of self-fulfilling prediction cannot therefore be completely excluded. Blinded outcome ascertainment remains an important methodological requirement for future external validation of the M.A.P.-RT.

### 2.3. Data Analysis

All analyses were performed in R (R Foundation for Statistical Computing version 4.5.2, Vienna, Austria), using the Boruta, glmnet, caret, pROC, lme4 and rsample packages. The analysis was conducted in three consecutive steps: first, feature selection was performed to identify the most informative M.A.P.-RT variables; second, predictive models were developed using the selected variables; finally, model performance was evaluated through internal validation procedures.

#### 2.3.1. Missing Data

Missing values on individual M.A.P.-RT items were infrequent and were replaced by the corresponding item median before the computation of the domain and total scores. No patient was excluded because of an incomplete assessment, and no imputation was applied to the outcome variables.

#### 2.3.2. Feature Selection

The Boruta algorithm, a wrapper around a random forest classifier, was applied to the full set of 28 individual M.A.P.-RT items together with the six domain scores and the Total Score (35 candidate variables), with a maximum of 500 runs. Boruta iteratively compares the importance of each real variable against that of randomised shadow copies and classifies variables as confirmed, tentative or rejected. Variable importance was summarised as the mean importance across iterations. This step is exploratory and descriptive. The six domain scores are sums of their constituent items and the Total Score is the sum of the six domains, so the 35 candidate variables are by construction mutually dependent rather than independent predictors. Boruta ranks each variable by the information it carries about the outcome relative to randomised shadow copies, but it does not partition variance between variables; the resulting importance estimates must therefore not be interpreted as independent contributions. The frequent confirmation of a domain score together with its own items, which is what we observed, reflects this shared variance and not additive predictive value. Accordingly, the Boruta ranking was used only to describe where the predictive information lies within the schedule, and no multivariable model reported below was specified on the basis of that ranking.

#### 2.3.3. Predictive Modelling

Elastic Net penalised logistic regression (α = 0.5, combining LASSO and Ridge penalties) was fitted on the six domain scores, which reduces overfitting and handles the substantial correlation among domains. The penalty parameter λ was selected by 10-fold cross-validation maximising the AUC, and the model was refitted at λ_min. To avoid perfect collinearity, the Total Score was not entered alongside the domain scores; its discriminative ability was assessed separately by ROC analysis. Odds ratios were obtained by exponentiating the model coefficients. For clinical interpretability, a standard non-penalised logistic regression with the same six predictors was additionally fitted to obtain odds ratios with 95% confidence intervals. Three considerations support building the multivariable models on the six domain scores rather than on the 28 individual items, despite Boruta having been run at item level. First, the domain is the unit at which the schedule is scored, interpreted and communicated within the multidisciplinary team, so a domain-level model is the one that can actually be used at the bedside. Second, with 61 sedation events, a six-predictor model remains within conventional events-per-variable limits, whereas a 28-item model would be substantially overfitted even under penalisation. Third, items within a domain are completed by a single professional and are strongly intercorrelated, so an item-level model would distribute one shared signal across collinear terms without improving either interpretability or performance. The item-level Boruta step was therefore retained as a descriptive check that the domains entered into the model were also those carrying most information at item level, and not as a variable-selection step feeding the regression.

#### 2.3.4. Internal Validation

Model performance was estimated by repeated 10 × 10-fold cross-validation. The dataset was partitioned into 10 folds stratified on the outcome; the procedure was fully nested. Within each outer training set of nine folds, λ was re-selected de novo by an inner 10-fold cross-validation restricted to that training set, the Elastic Net was refitted at the resulting λ_min, and the model was then applied to the held-out fold, which contributed no information to either variable standardisation or penalty selection. No tuning was performed on the full dataset before cross-validation, so no information leakage occurred during model optimisation. The λ values quoted in the Results refer to the descriptive model fitted to the complete dataset and were never used to generate the cross-validated predictions. The procedure was repeated with ten different random fold allocations, and for each patient, the ten out-of-fold predicted probabilities were averaged into a single estimate. Discrimination was summarised by the AUC with DeLong 95% confidence intervals. The Youden index identified the optimal probability threshold, and classification performance at that threshold was described by accuracy, sensitivity, specificity, positive and negative predictive values and Cohen’s κ. McNemar’s test assessed asymmetry between false-positive and false-negative rates. Accuracy was compared with the no-information rate by exact binomial test.

For both outcomes, the clinically relevant class was treated as the positive class—that is, “sedation required” and “intensive psychological support required”.

Finally, linear regression models were used to investigate whether age- and gender-related profiles could predict psychological distress and difficulties potentially affecting adaptation throughout the radiotherapy pathway. Two age groups were defined: children (≤11 years) and adolescents and young adults (AYA; ≥12 years) [19]. This cut-off was pre-specified on developmental and service-organisation grounds and was not derived from the present data: 12 years is the conventional lower bound of the AYA age range in paediatric oncology; it approximates the transition to formal operational reasoning about illness, bodily integrity and time, and it is the point at which our own service moves from play-based to interview-based preparation. No data-driven search for an optimal cut-off was performed, and age was not additionally modelled as a continuous variable in these analyses. Each dependent variable was analyzed using a separate linear model including age group, gender, and their interaction as predictors. In every model, the AYA group and male sex were the reference categories, so a positive coefficient denotes a higher score in children and in females, respectively. Statistical significance was set at *p* < 0.05. Because each outcome represented a distinct clinical domain, the models were specified and fitted independently. However, because fifteen outcome models were fitted, the *p* values for the age-group effect were additionally adjusted across this family of tests using the Benjamini–Hochberg false-discovery-rate procedure; unadjusted and adjusted values are reported together in Table 1 and in Supplementary Table S1.

The objective was to identify patient subgroups at increased risk of psychological difficulties, thereby facilitating early detection of vulnerable patients and the implementation of tailored adaptation strategies and supportive care over time.

## 3. Results

### 3.1. Study Population

Of 270 consecutive patients enrolled, 268 were included in the analysis after exclusion of two age outliers. The final cohort ranged in age from 1 to 23 years (mean 9.6, SD 5.7) and comprised 159 male (59.3%) and 109 female (40.7%) patients. Central nervous system tumours were the most frequent diagnostic group (118 patients, 44.0%), followed by haematological malignancies (80, 29.9%), sarcomas (33, 12.3%), other diagnoses (20, 7.5%) and neuroblastoma (17, 6.3%). Of the analytic sample, 169 patients (63.06%) were children aged 11 years or younger, and 99 (36.94%) were adolescents and young adults aged 12 years or older. Patient characteristics, including the distribution of age, treatment modality, sedation status and psychological-support category, are summarised in Table 2.

Sedation was required during radiotherapy by 61 patients (22.8%), while 207 (77.2%) completed treatment without it. According to treatment type, sedation was required in 16 of 49 patients (32.7%) undergoing craniospinal irradiation, 14 of 52 patients (26.9%) receiving total body irradiation (TBI), and 31 of 167 patients (18.6%) treated with standard radiotherapy. With respect to the secondary outcome, 137 patients (51.1%) received an intensive course of psychological support and 131 (48.9%) received standard support.

The M.A.P.-RT schedule was completed for all patients. Mean domain scores (and observed ranges) were: Score A 5.9 (0–10), Score B 3.3 (0–10), Score C 8.5 (0–18), Score D 11.9 (3–26), Score E 13.7 (5–25), Score F 16.7 (5–27), and Total Score 60.0 (17–105).

### 3.2. Prediction of the Need for Sedation

#### 3.2.1. Feature Selection Results

**Figure 1:**
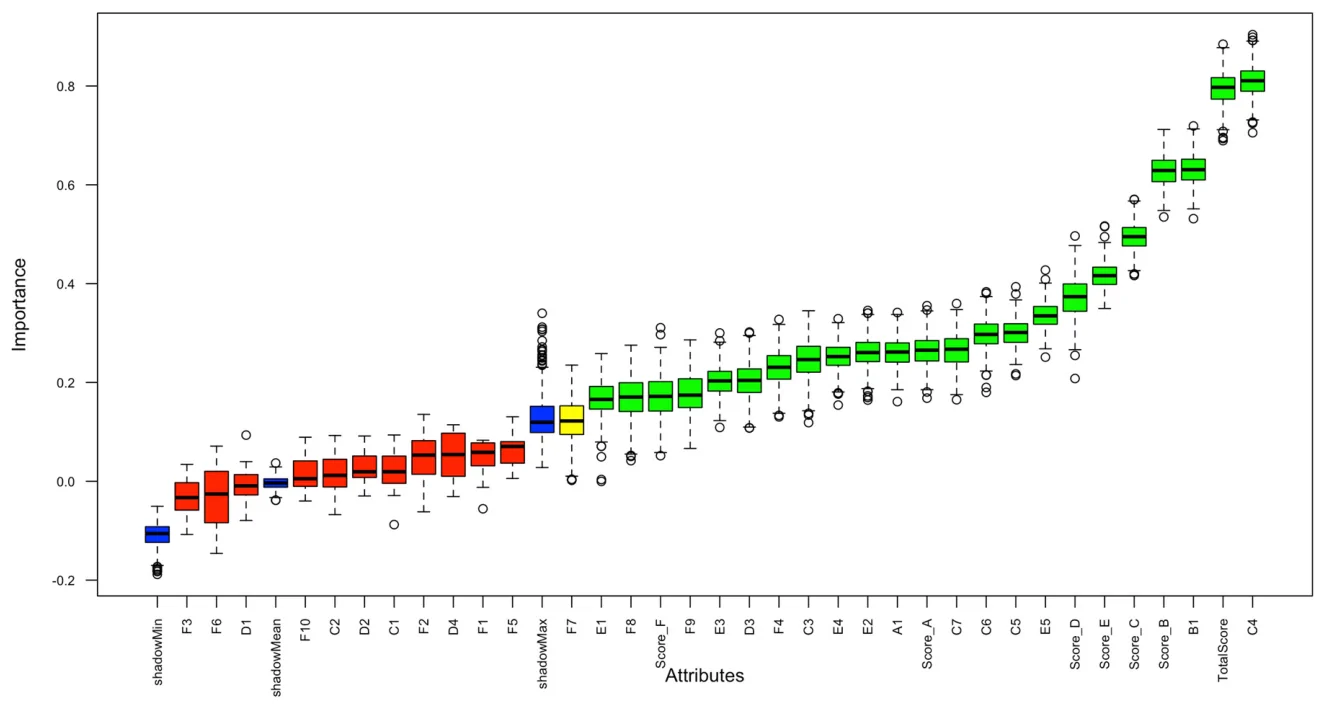
Boruta feature selection for the prediction of the need for sedation. Boxplots show the distribution of importance scores for each candidate variable (single M.A.P.-RT items, the six partial scores A–F, and the Total Score) across 499 algorithm iterations. Green boxes indicate variables confirmed as significant predictors, red boxes indicate variables rejected as not predictive, and yellow boxes indicate tentative variables for which no definitive decision was reached. Blue boxes (shadowMin, shadowMean, shadowMax) represent the minimum, mean, and maximum importance of the randomized shadow attributes used as the reference threshold by the algorithm. The circles represent outliers.

Boruta confirmed 23 of the 35 candidate variables as predictive of the need for sedation, rejected 11, and left one—F7, parental anxiety—tentative (Figure 1). All six domain scores and the Total Score were confirmed, together with 16 individual items: A1, B1, C3, C4, C5, C6, C7, D3, E1, E2, E3, E4, E5, F4, F8 and F9. Ranked by mean importance, the strongest predictors were collaboration during previous radiographic procedures (C4, 0.813), the Total Score (0.799), age-related discomfort (B1, 0.628) and its corresponding domain score (Score B, 0.627), followed by Score C (0.497), Score E (0.414) and Score D (0.370).

The pattern of confirmed items is clinically coherent: it combines the developmental dimension, the collaboration and distress items recorded by the radiation oncologist at first evaluation (C3–C7), and the full set of CEMS behavioural observations made on first entry to the treatment room (E1–E5). By contrast, items concerning the information held by the family and the patient (C1, C2), most physical items (D1, D2, D4) and the majority of psychological interview items (F1, F2, F3, F5, F6, F10) were rejected.

#### 3.2.2. Multivariable Models

The Elastic Net model (λ_min = 0.0039) retained all six domain scores with non-zero coefficients (Table 3). Score B carried by far the largest effect (OR 1.65), followed by Score C (OR 1.30), Score D (OR 1.13), Score E (OR 1.08), Score A (OR 1.07) and Score F (OR 1.04). In the non-penalised logistic model fitted for clinical interpretability, three domains were independently and significantly associated with the need for sedation: Score B (OR 1.76, 95% CI 1.41–2.29, *p* < 0.001), Score C (OR 1.34, 95% CI 1.08–1.70, *p* = 0.010) and Score D (OR 1.16, 95% CI 1.06–1.28, *p* = 0.001). Score A, Score E and Score F did not reach statistical significance.

#### 3.2.3. Predictive Performance

**Figure 2:**
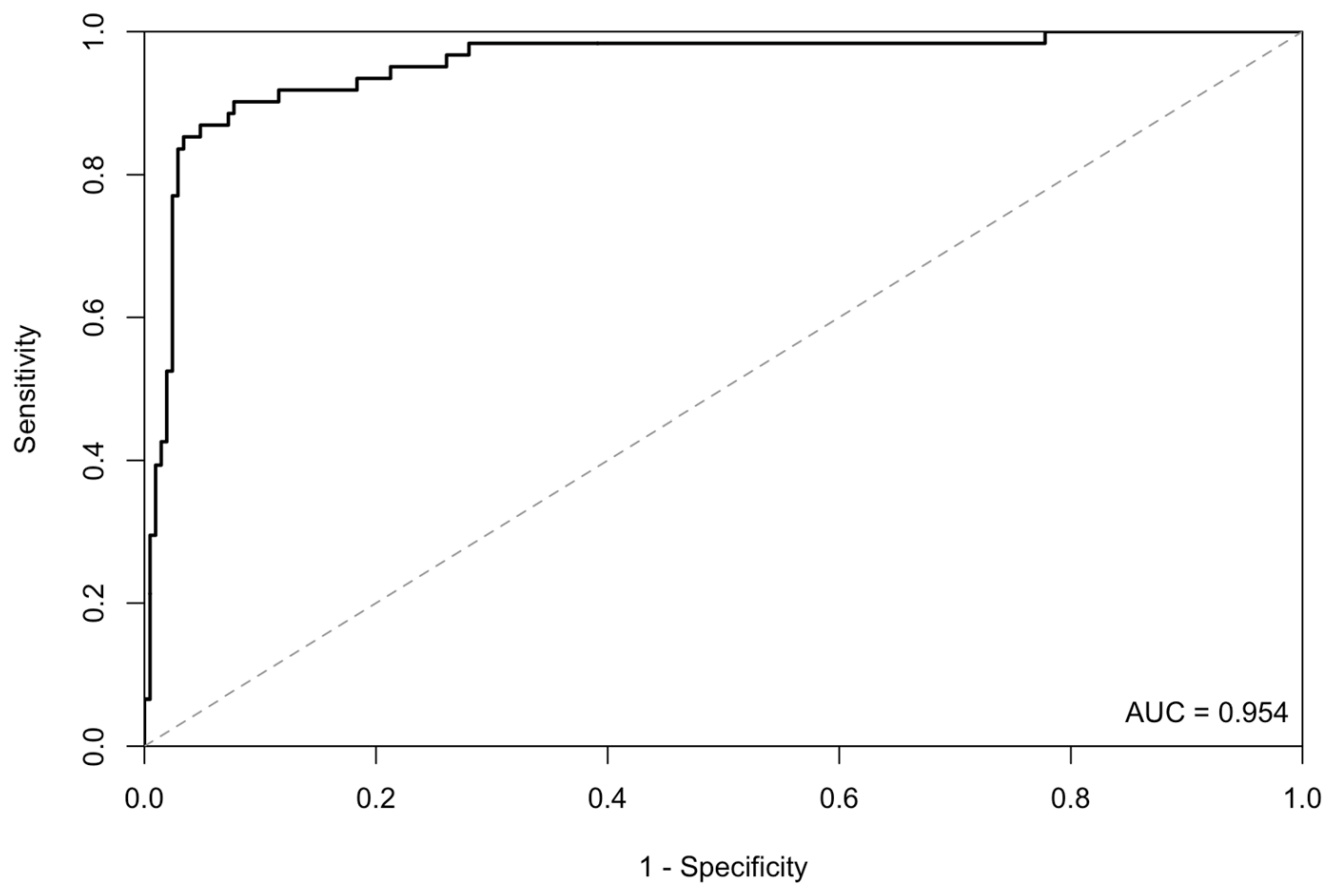
Cross-validated ROC curve for the prediction of the need for procedural sedation or general anaesthesia (AUC = 0.954, 95% CI 0.922–0.986). The curve (solid line) is derived from the out-of-fold predicted probabilities of the Elastic Net model fitted to the six M.A.P.-RT domain scores (A–F) under repeated nested 10 × 10-fold cross-validation; for each patient, the ten out-of-fold probabilities (100 model fits in total) were averaged into a single estimate. The dashed line represents the line of random classification.

Repeated 10 × 10-fold cross-validation generated 100 out-of-fold probability estimates per patient, averaged into a single prediction for each of the 268 patients. Discrimination was excellent, with an AUC of 0.954 (95% CI 0.922–0.986) (Figure 2). At the Youden-optimal threshold (predicted probability 0.33), sensitivity was 0.90 and specificity 0.92; the confusion matrix comprised 55 true positives, 191 true negatives, 16 false positives and 6 false negatives.

Overall accuracy was 0.918 (95% CI 0.878–0.948), significantly above the no-information rate of 0.772 (*p* = 2.5 × 10^−10^). Positive and negative predictive values were 0.77 and 0.97, respectively, and Cohen’s κ was 0.78, indicating substantial agreement between predicted and observed outcomes. McNemar’s test did not show significant asymmetry between the two error types (*p* = 0.055), suggesting that misclassifications were reasonably balanced.

#### 3.2.4. Total Score Alone

The composite Total Score, used without penalised regression, achieved an AUC of 0.940 (95% CI 0.901–0.979), with sensitivity of 0.92 and specificity of 0.88 at the Youden-optimal cut-off of 71.5. Both approaches therefore discriminated well; the cross-validated Elastic Net model achieved a modestly higher AUC (0.954 versus 0.940) and a higher specificity, corresponding to fewer children incorrectly flagged as requiring sedation.

### 3.3. Prediction of the Intensity of Psychological Support

In contrast to the strong performance observed for sedation, prediction of the intensity of psychological support was considerably weaker. Boruta confirmed only 7 of the 35 candidate variables, led by the minutes required for the radiotherapy fraction (D4, mean importance 0.531) and by the physical-skills domain that contains it (Score D, 0.517), followed by the Total Score, Score E, distress at the first medical evaluation (C7), the nursing pain/distress rating (A1) and Score A. In the non-penalised logistic model, Score D (OR 1.10, 95% CI 1.05–1.15, *p* < 0.001) and Score A (OR 1.27, 95% CI 1.08–1.51, *p* = 0.006) were independently associated with the need for intensive support, while Score B showed a non-significant trend (OR 1.11, 95% CI 0.99–1.26, *p* = 0.083). Under the same repeated nested 10 × 10-fold cross-validation, discrimination was fair: AUC of 0.714, sensitivity of 0.81, specificity of 0.50, positive predictive value of 0.63, negative predictive value of 0.72, overall accuracy of 0.660 (95% CI 0.600–0.717) against a no-information rate of 0.511 (*p* = 5.5 × 10^−7^), and Cohen’s κ of 0.32. McNemar’s test showed a significant asymmetry between error types (*p* < 0.001): the model predicted intensive support for 176 patients against the 137 who actually received it, and therefore systematically over-classified patients into the higher-need category. The corresponding Boruta plot, ROC curve and full coefficient table are reported in Supplementary Figures S1 and S2.

### 3.4. Age and Gender as Predictors of Psychological Difficulties and Anxiety-Related Outcomes

Age group was the only demographic variable associated with psychosocial vulnerability. Children ≤ 11 years scored higher than adolescents and young adults on psychological difficulties, distress reactive to diagnosis, patient and parental fear/anxiety, externalising coping, parenting difficulties and family/social/work difficulties, and on all five CEMS subscales recorded on first entry to the treatment room. No effect was found for recent loss of autonomy, internalising coping or traumatic events before diagnosis. Sex was not significant in any model, nor was the age group × sex interaction. All effects that were significant before multiplicity correction remained significant after Benjamini–Hochberg adjustment (Table 1 and Table S1), although the margin was narrow for psychological difficulties (adjusted *p* = 0.044). Explained variance was modest for the psychological-interview items with small coefficients (R^2^ 0.036 to 0.053) and substantially larger for externalising coping, parenting difficulties and the CEMS subscales (R^2^ 0.222 to 0.297), a distinction taken up in the Discussion.

Overall, age group emerged as the main predictor of distress-related outcomes. Moreover, younger children showed greater psychological, emotional, and behavioural difficulties upon entering the LINAC room compared with adolescents and young adults, whereas no significant differences emerged between sexes or for age group × sex interactions (for a visualization, see Figures S3–S14). These findings suggest that younger patients may represent a more vulnerable group requiring targeted support to improve treatment adaptation.

## 4. Discussion

Radiotherapy for pediatric cancer requires the child to lie still, alone, inside the treatment machine daily for several weeks, a demand made harder by prior painful procedures, unfamiliar staff, and disruption to family routine. When distress exceeds what the child can tolerate, procedural sedation or general anaesthesia becomes necessary, but it is burdensome: it requires fasting, dedicated staff, and extended room time, and the SIOP Europe Working Group’s recent review found that shared protocols are still lacking across institutions [4,5]. As reviewed in the Introduction, preparation rather than sedation can meet the child’s needs in a substantial proportion of cases [6,7,8,9,10,20,21,22,23,24]. Two findings from that literature bear directly on how a screening instrument should be used. First, tailoring matters: a preparatory kit well received by younger children was not accepted by adolescents, who wanted individualized, interactive solutions [25]. Second, the family, rather than the child alone, is the effective unit of intervention [26,27]. These programmes are effective but resource-intensive, and the professionals who deliver them are scarce in an already highly specialized team [28,29]. Offering intensive preparation to everyone is not feasible; defaulting to sedation for every young child exposes many to an unnecessary intervention. The M.A.P.-RT schedule was designed to identify, at first contact, which children need which pathway [18], and the present study asked whether the pilot’s results would hold in a substantially larger prospective cohort.

In a sample more than two and a half times the size of the original, and under a considerably more demanding internal validation, in which every prediction concerned a patient unseen during model fitting, the schedule again predicted the need for sedation with excellent discrimination (AUC 0.954, 95% CI 0.922–0.986).

More important than accuracy itself is that the predictive items proved stable. Age (group B) was confirmed as fundamental, but the schedule is not merely a proxy for it: developmental information retained its predictive strength alongside medical, behavioural and psychological data, capturing differences between same-age children that a purely age-based rule would miss. This is relevant given how narrowly age-banded the preparation programmes discussed above were [9,20,21,22]. The group C items collected at first medical evaluation were again among the most informative, led by the child’s collaboration during previous radiographic procedures and a natural and cost-free rehearsal of what radiotherapy will require, together with collaboration with staff requests, tolerance of separation from the parent, and distress at that visit. The prominence of the separation item echoes the centrality of the family in both Filin’s and Fortier’s work [20,26]. The entire group E block, comprising the CEMS observations made as the child first enters the treatment room, was likewise confirmed; these take seconds to record and require no extra resources, and just as Engvall’s children described that visit as clarifying [25], our data suggest it is equally clarifying for the observing team.

Prediction of psychological-support intensity was considerably weaker, misclassifying roughly one patient in three, with a significant bias toward over-classifying patients into the higher-need category rather than under-classifying them. Here, the informative items were not the developmental and behavioural indicators that predicted sedation, but the practical burden of treatment, chiefly fraction duration and the physical-skills domain, together with distress recorded at first contact. The two outcomes are thus driven by different vulnerabilities. We believe this reflects a deeper distinction: sedation need is generally determined by characteristics observable before treatment or early in the treatment pathway, whereas support intensity, which in our protocol consists of weekly sessions escalating to several per week or daily for complex or crisis cases [16], evolves with fatigue, side-effects and acute crises over the course of treatment. No single-occasion assessment, however thorough, can capture that trajectory. The M.A.P.-RT should therefore trigger psychological evaluation for children whose profile suggests elevated need, rather than substitute for the psychologist’s ongoing judgement, following the same logic that underlies the Psychosocial Assessment Tool’s use elsewhere in pediatric oncology [12,14,15]. It is worth stating explicitly why a single multidimensional instrument should perform so differently across two outcomes it was designed to address. Two reasons converge. First, the two outcomes differ in temporal structure: the need for sedation is decided once, essentially before treatment begins, and is largely determined by characteristics of the child that are already present and observable at the first consultation, whereas the amount of psychological support delivered accumulates over five to six weeks and responds to events that have not yet occurred. A baseline measurement can predict a baseline-determined outcome; it cannot predict a trajectory. Second, they differ in how sharply they are defined: sedation is a categorical, verifiable clinical act with an unambiguous criterion, while the intensity of psychological support is a continuous quantity dichotomised at an administrative threshold, so patients close to that threshold are assigned to classes that differ by a single session. Measurement error at the boundary is therefore built into the outcome itself. The asymmetry is thus not a defect of the instrument but a property of the two targets, and it justifies the different clinical uses we propose for them.

Beyond prediction, the schedule gives the radiation oncologist, nurse, technician, psychologist and residents a quick, shared language for describing the child at first consultation, making explicit what each has observed and letting the team plan preparation accordingly.

These results also speak to a broader argument for embedding structured profiling, rather than clinical impression alone, into the radiotherapy pathway. In the absence of an a priori assessment, teams face two default options, sedating cautiously to avoid the risk of an uncooperative session, or asking every child to attempt treatment awake. Both are blunt instruments. The first exposes children who would have coped without anesthesia to an avoidable intervention; the second risks distress, physical resistance and, because accurate beam delivery depends on sub-millimetric immobilisation throughout every fraction, a treatment-geometry error driven by an unanticipated panic response. Profiling replaces this binary default with a graded allocation of preparation resources, consistent with the matched-care logic already established for family psychosocial risk more broadly, and increasingly promoted within paediatric oncology networks that call for radiotherapy to be embedded in a global, multidisciplinary model of care rather than treated as a purely dosimetric problem. Our age-related findings reinforce this point: because younger children showed systematically greater vulnerability across nearly every psychological, coping and CEMS behavioural dimension examined, age functions as a coarse but immediately available profiling variable, evident before a full M.A.P.-RT assessment can even be completed, that could itself trigger closer procedural attention while the fuller assessment is underway.

### 4.1. Strengths and Limitations

The main methodological strengths of this work are its prospective design with consecutive enrolment; a sample more than two and a half times larger than the pilot, with 61 sedation events comfortably within conventional events-per-variable limits for a six-predictor model; multidisciplinary rather than single-rater assessment; a penalised model that tolerates the collinearity among domains; and fully nested repeated cross-validation, in which every reported prediction concerns a patient unseen during both feature scaling and penalty selection. Clinically, the instrument is completed at a consultation that already takes place and yields an estimate before the first treatment fraction, when preparation can still be arranged.

The limitations are that this was an observational study without randomisation, so associations cannot be read causally. Validation was internal: repeated cross-validation is more rigorous than apparent performance but not a substitute for external validation, and results may prove optimistic in centres with different sedation policies, staffing and thresholds for psychological support, given the documented variation across European pediatric radiotherapy centres [4,30]. Our case-mix, dominated by CNS and haematological malignancies, may not generalize. The psychological-support threshold was inherited from the pilot rather than clinically validated, and the team completing the schedule also participated in the sedation and support decisions, so incorporation bias, although low, cannot be excluded. Formal inter-rater reliability and feasibility data (completion time, workload) were not collected, a particular concern for the observation-based domains A and E. Enrolment spanned 2019–2025, including the COVID-19 pandemic, and secular change in psychosocial variables over this window cannot be excluded. Finally, fifteen age- and sex-related models were fitted; Benjamini–Hochberg correction left all significant effects significant (see Table S1), but several had modest explained variance (R^2^ as low as 0.036) and should be interpreted cautiously, alongside the study’s single-centre design.

### 4.2. Future Directions

The priority is external validation in independent centres, ideally with prospective registration. Translating the schedule into a digital format integrated into the clinical workflow would facilitate both administration and the multicentre data collection that validation requires. Finally, the logical endpoint of this line of research is an interventional design in which M.A.P.-RT stratification is used prospectively to allocate preparation resources, with anesthesia use as the primary outcome, the step that would establish not merely that the instrument predicts, but that acting on its predictions changes practice.

## 5. Conclusions

In a prospective cohort of 268 children and adolescents, the M.A.P.-RT schedule confirmed the excellent predictive accuracy for the need for procedural sedation reported in the original RADAR study, with stable predictors and substantially improved precision (cross-validated AUC of 0.954, 95% CI of 0.922–0.986; sensitivity of 0.90, specificity of 0.92, positive predictive value of 0.77, negative predictive value of 0.97, Cohen’s κ of 0.78). Developmental stage, the first medical evaluation and the physical skills required for treatment carried independent predictive weight, and the simple Total Score performed almost as well as the full multivariable model. Prediction of the intensity of psychological support was considerably weaker and rested on a different set of dimensions, indicating that the two supportive-care needs are distinct and that the instrument is best used as a screening trigger in that domain. Given that psychological needs may change throughout the treatment trajectory, a longitudinal assessment approach could provide valuable insights into patients’ evolving experiences. Repeating selected sections of the M.A.P.-RT (particularly Domains E and F) at clinically relevant time points may enable the detection of emerging psychological distress and relational difficulties that are not identifiable during the initial assessment, thereby supporting timely and tailored interventions.

Administered once, at the first multidisciplinary consultation, the M.A.P.-RT provides a structured basis for anticipating which children have an increased likelihood of requiring sedation and for directing procedural preparation towards those most likely to benefit. Incorporating a final assessment at the end of radiotherapy could further strengthen the clinical value of the M.A.P.-RT, not only by allowing confirmation of the initial predictive performance but also by providing an outcome measure to evaluate whether the psychological interventions triggered by the screening process effectively improve patients’ adaptation and overall treatment experience. Finally, having been developed, applied and validated within a single paediatric radiotherapy programme with internal validation, the M.A.P.-RT now provides a well-characterised starting point for prospective testing in independent centres with different sedation policies, staffing models and thresholds for psychological support.

## Figures and Tables

**Table 1:** Linear models of age group, sex and their interaction for the ten items of the psychological interview (F1–F10) and the five CEMS subscales (E1–E5). Coefficients refer to the age-group effect; the reference categories are the AYA group (≥12 years) and male sex, so a positive β denotes a higher score in children ≤ 11 years. All models were fitted with 3 and 264 degrees of freedom. *p* (BH) is the Benjamini–Hochberg false-discovery-rate-adjusted value across the 15 models.

Outcome	β (Children vs. AYA)	95% CI	t	*p*	*p* (BH)	R^2^
Psychological difficulties (F1)	0.35 (SE 0.17)	0.02 to 0.67	2.11	0.036	0.044	0.036
Recent loss of autonomy (F2)	−0.05 (SE 0.16)	−0.36 to 0.26	−0.30	0.764	0.764	0.012
Distress reactive to diagnosis (F3)	0.33 (SE 0.11)	0.11 to 0.56	2.93	0.004	0.006	0.040
Externalising coping (F4)	1.24 (SE 0.17)	0.90 to 1.58	7.22	<0.001	<0.001	0.246
Internalising coping (F5)	−0.19 (SE 0.13)	−0.45 to 0.08	−1.38	0.169	0.195	0.018
Patient fear/anxiety (F6)	0.33 (SE 0.11)	0.12 to 0.54	3.12	0.002	0.004	0.045
Parental fear/anxiety (F7)	0.29 (SE 0.10)	0.10 to 0.49	2.94	0.004	0.006	0.039
Parenting difficulties (F8)	1.07 (SE 0.16)	0.75 to 1.39	6.56	<0.001	<0.001	0.222
Family/social/work difficulties (F9)	0.38 (SE 0.15)	0.09 to 0.67	2.57	0.011	0.015	0.053
Traumatic events before diagnosis (F10)	0.09 (SE 0.18)	−0.26 to 0.45	0.52	0.602	0.645	0.005
CEMS facial expression (E1)	1.13 (SE 0.16)	0.81 to 1.44	7.11	<0.001	<0.001	0.227
CEMS vocalisation (E2)	1.56 (SE 0.20)	1.17 to 1.95	7.87	<0.001	<0.001	0.236
CEMS activity (E3)	1.60 (SE 0.22)	1.16 to 2.04	7.16	<0.001	<0.001	0.249
CEMS interaction (E4)	1.75 (SE 0.21)	1.35 to 2.16	8.49	<0.001	<0.001	0.297
CEMS cooperation (E5)	1.66 (SE 0.20)	1.27 to 2.06	8.27	<0.001	<0.001	0.272

CI = confidence interval; CEMS = Children’s Emotional Manifestation Scale.

**Table 2:** Descriptive statistics and clinical characteristics of the study population (N = 268).

**Sex**	**N (%)**
Male	159 (59.33%)
Female	109 (40.67%)
**Age**	Mean 9.6, SD 5.7, range 1–23
Children ≤ 11 years	169 (63.06)
Adolescents and young adults ≥ 12 years	99 (36.94)
**Histological Diagnosis**	**N (%)**
Central Nervous System Neoplasm	118 (44.03%)
Hematological Neoplasm	80 (29.85%)
Sarcoma	33 (12.31%)
Others (e.g., Hepatocarcinoma, Nephroblastoma, Retinoblastoma)	20 (7.46%)
Neuroblastoma	17 (6.34%)
**Treatment modality**	**N (%)**
Focal radiotherapy	167 (62.31%)
Total body irradiation	52 (19.40%)
Craniospinal irradiation	49 (18.28%)
**Procedural sedation**	**N (%)**
Required	61 (22.76%)
Not required	207 (77.24%)
**Psychological support**	**N (%)**
Intensive	137 (51.12%)
Standard	131 (48.88%)
**M.A.P.-RT scores**	**Mean (observed range)**
Score A/B/C	5.9 (0–10)/3.3 (0–10)/8.5 (0–18)
Score D/E/F	11.9 (3–26)/13.7 (5–25)/16.7 (5–27)
Total Score	60.0 (17–105)

**Table 3:** Associations between the six M.A.P.-RT domain scores and the two outcomes. For each outcome, the penalised (Elastic Net, α = 0.5) odds ratio is reported alongside the odds ratio, 95% confidence interval and *p* value from the non-penalised logistic model fitted with the same six predictors for clinical interpretability. Odds ratios are expressed per one-point increase in the domain score. The Elastic Net retains all predictors with non-zero coefficients and does not provide confidence intervals, which is why both models are shown.

Domain	Sedation: EN OR	Sedation: Logistic OR (95% CI)	Sedation: *p*	Intensive Support: EN OR	Intensive Support: Logistic OR (95% CI)	Intensive Support: *p*
A—Pain/distress (nursing)	1.07	1.08 (0.80–1.48)	0.612	1.22	1.27 (1.08–1.51)	0.006
B—Development/age	1.65	1.76 (1.41–2.29)	<0.001	1.10	1.11 (0.99–1.26)	0.083
C—First medical evaluation	1.30	1.34 (1.08–1.70)	0.010	0.95	0.90 (0.79–1.03)	0.135
D—Physical skills for RT	1.13	1.16 (1.06–1.28)	0.001	1.10	1.10 (1.05–1.15)	<0.001
E—Emotional distress (CEMS)	1.08	1.07 (0.96–1.20)	0.229	1.05	1.07 (0.99–1.17)	0.089
F—Psychological interview	1.04	1.06 (0.91–1.23)	0.450	0.94	0.94 (0.86–1.02)	0.120

EN = Elastic Net.

## Data Availability

The raw data supporting the conclusions of this article will be made available by the authors on request. Sharing the data could potentially compromise the privacy of the participants, as stipulated in the ethics approval. Requests to access the datasets may be considered on a case-by-case basis, subject to review and approval by the authors and the appropriate ethics board.

## Supplementary Materials

The following supporting information can be downloaded at: https://www.mdpi.com/article/10.3390/children13101328/s1, Figure S1: Boruta feature selection for the prediction of the intensity of psychological support (standard vs. intensive); Figure S2: Plot of ROC curve (solid line) for the “psychological support” model; Figure S3: Estimated marginal means of Vocalization according to age group and gender; Figure S4: Estimated marginal means of Interaction according to age group and gender; Figure S5: Estimated marginal means of Facial Expression according to age group and gender; Figure S6: Estimated marginal means of Cooperation according to age group and gender; Figure S7: Estimated marginal means of Activity according to age group and gender; Figure S8: Estimated marginal means of Psychosocial Difficulties according to age group and gender; Figure S9: Estimated marginal means of Post Diagnosis Distress according to age group and gender; Figure S10: Estimated marginal means of Parental Anxiety according to age group and gender; Figure S11: Estimated marginal means of Patient Management Difficulties according to age group and gender; Figure S12: Estimated marginal means of Fears and Anxiety according to age group and gender; Figure S13: Estimated marginal means of Family and Work Difficulties according to age group and gender; Figure S14: Estimated marginal means of Externalising Coping Style according to age group and gender; Table S1: Benjamini–Hochberg false-discovery-rate correction applied to the age-group effect across the 15 linear model.
