# Supplementary material for: Enhancing Decision-Making in Pediatric Radiotherapy: Internal Validation and Expansion of the M.A.P.-RT Schedule for Sedation Need Prediction and Identification of Psychological Support Needs in a Prospective Cohort of 268 Pediatric Patients
Source: Children (Basel). 2026 Sep 30;13(10):1328. doi: 10.3390/children13101328 (PMC13702979; doi:10.3390/children13101328)
Supplement: Supplementary file 1 [file children-13-01328-s001.zip › children-4525224-supplementary.pdf]

Prediction of the intensity of psychological support

Feature selection. For the secondary outcome, Boruta confirmed a much smaller set of variables: 7 of 35 were confirmed, 5 were tentative and 23 were rejected (Figure S1). The confirmed variables were, in order of mean importance, D4 (minutes required for the RT fraction, 0.531), Score D (0.517), the Total Score (0.339), Score E (0.249), C7 (distress detected during the first medical evaluation, 0.216), A1 (nursing pain/distress, 0.166) and Score A (0.164). The tentative group comprised E5, Score B, B1, E1 and E2.

This profile differs substantially from that observed for sedation. Where sedation was driven by developmental stage and by medical and behavioural indicators of collaboration, the intensity of psychological support was dominated by the practical burden of treatment, the time required per fraction and the physical skills domain that contains it — together with distress recorded at first contact.

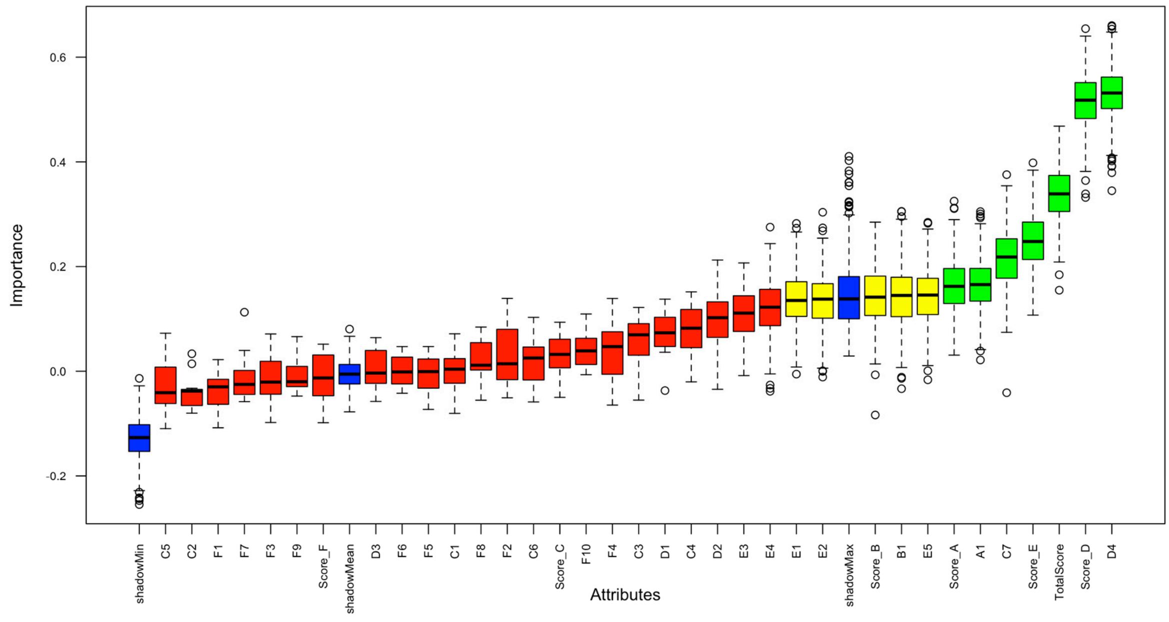

Figure S1. Boruta feature selection for the prediction of the intensity of psychological support (standard vs. intensive). Boxplots show the distribution of importance scores for each candidate variable (single M.A.P.-RT items, the six partial scores A–F, and the Total Score) across 499 algorithm iterations. Green boxes indicate variables confirmed as significant predictors, red boxes indicate variables rejected as not predictive, and yellow boxes indicate tentative variables for which no definitive decision was reached. Blue boxes (shadowMin, shadowMean, shadowMax) represent the minimum, mean,

and maximum importance of the randomized shadow attributes used as the reference threshold by the algorithm. The circles represent outliers.

Multivariable models. In the Elastic Net model (λ_min = 0.0048), Score A carried the largest positive coefficient (OR 1.22), followed by Score B (OR 1.10), Score D (OR 1.10) and Score E (OR 1.05); Score C and Score F showed small negative coefficients (OR 0.95 and 0.94). In the non-penalised logistic model, expressed with intensive support as the outcome of interest, Score D was the strongest independent predictor (OR 1.10, 95% CI 1.05–1.15, $p < 0.001$) and Score A was also significantly associated with the need for intensive support (OR 1.27, 95% CI 1.08–1.51, $p = 0.006$); Score B showed a non-significant trend (OR 1.11, 95% CI 0.99–1.26, $p = 0.083$), while Score C, Score E and Score F were not significant.

Predictive performance. Repeated cross-validation yielded an AUC of 0.714 (Figure S2), substantially below that obtained for sedation. Treating "intensive support required" as the positive class, the model correctly identified 111 of the 137 patients who received intensive support (sensitivity 0.81) but only 66 of the 131 who received standard support (specificity 0.50). Positive and negative predictive values were 0.63 and 0.72 respectively. Overall accuracy was 0.660 (95% CI 0.600–0.717), significantly above the no-information rate of 0.511 ($p = 5.5 \times 10^{-7}$), but agreement was only fair (Cohen's $\kappa = 0.32$). McNemar's test revealed significant asymmetry between error types ($p < 0.001$): the model predicted intensive support for 176 patients against the 137 who actually received it, and therefore systematically over-classified patients into the higher-need category.

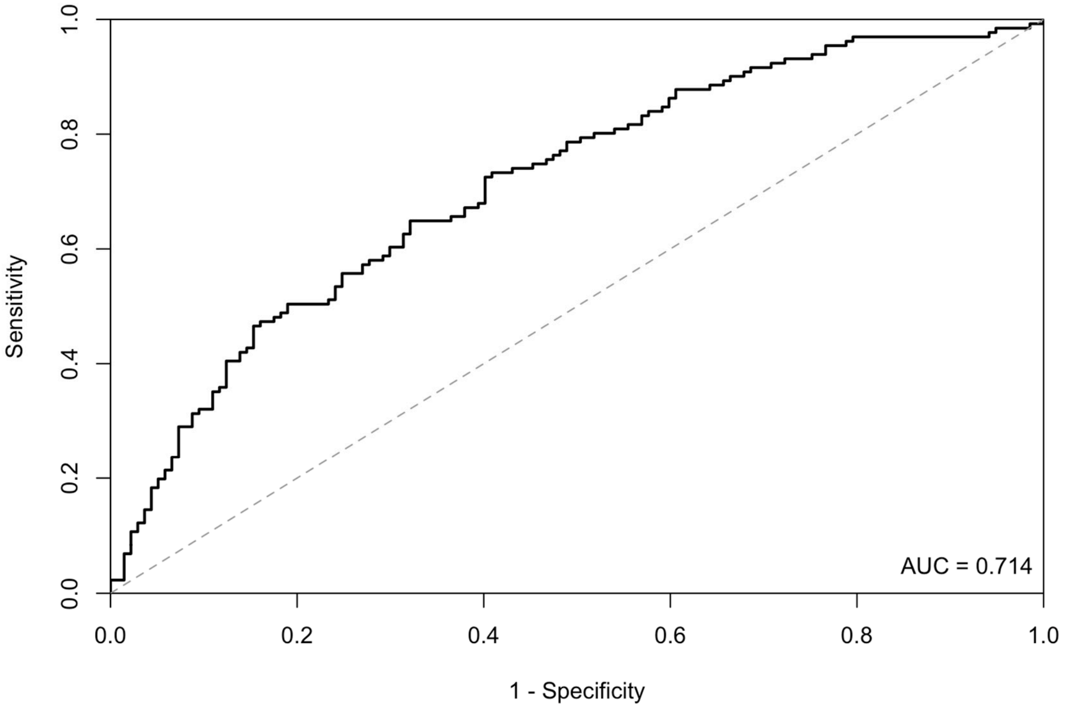

Figure S2. Plot of ROC curve (solid line) for the "psychological support" model (AUC= 0.714), showing possibility to identify patients who need intensive psychological support with regards to the six domain scores, achieved putting into the model the value of relevant items of M.A.P.-RT. The dashed line represents the line of random classification.

Figures S3–S14 show the estimated marginal means of each outcome by age group and gender, illustrating the age-group effects reported in Table 3 and the absence of any sex or interaction effect.

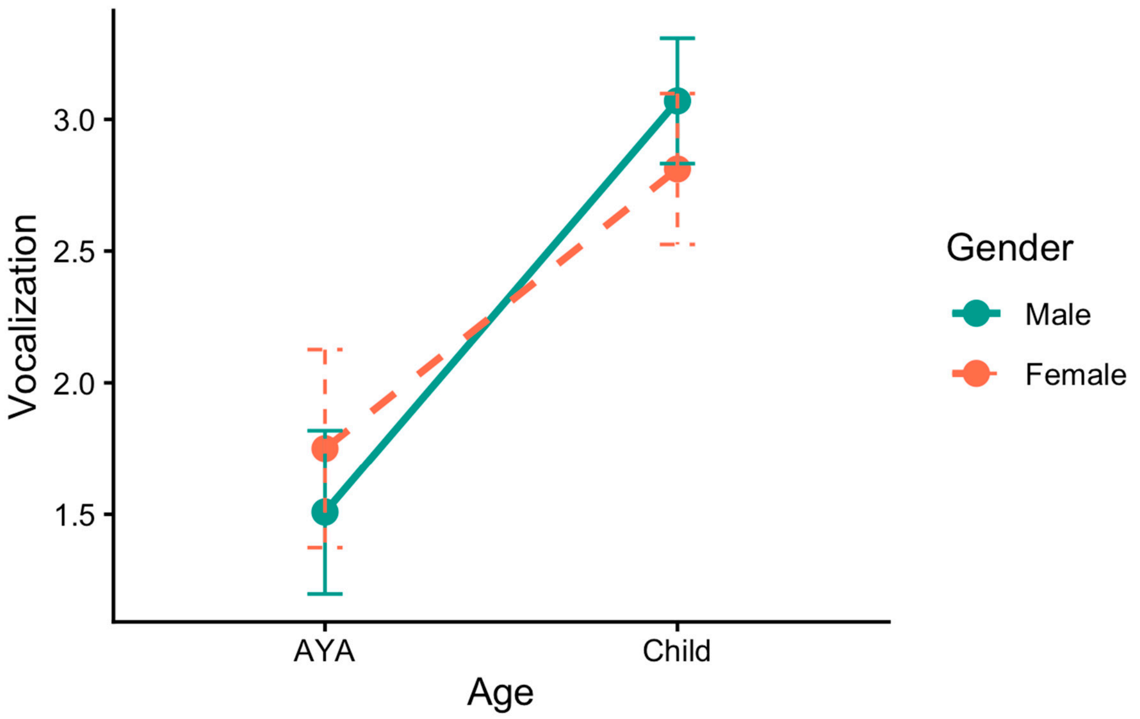

Figure S3. Estimated marginal means of Vocalization according to age group and gender. The plot illustrates the interaction between age group and gender derived from the linear regression model, with points representing model-estimated mean scores and error bars indicating 95% confidence intervals. Solid and dashed lines represent males and females, respectively.

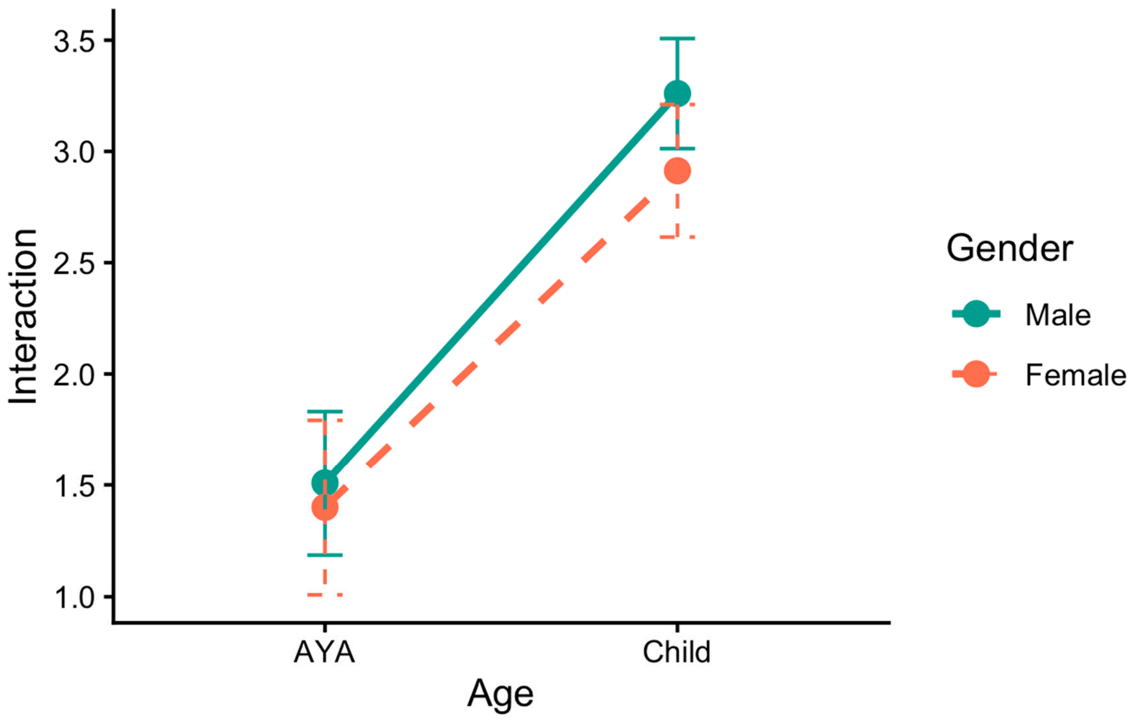

Figure S4. Estimated marginal means of Interaction according to age group and gender. The plot illustrates the interaction between age group and gender derived from the linear regression model, with points representing model-estimated mean scores and error bars indicating 95% confidence intervals. Solid and dashed lines represent males and females, respectively.

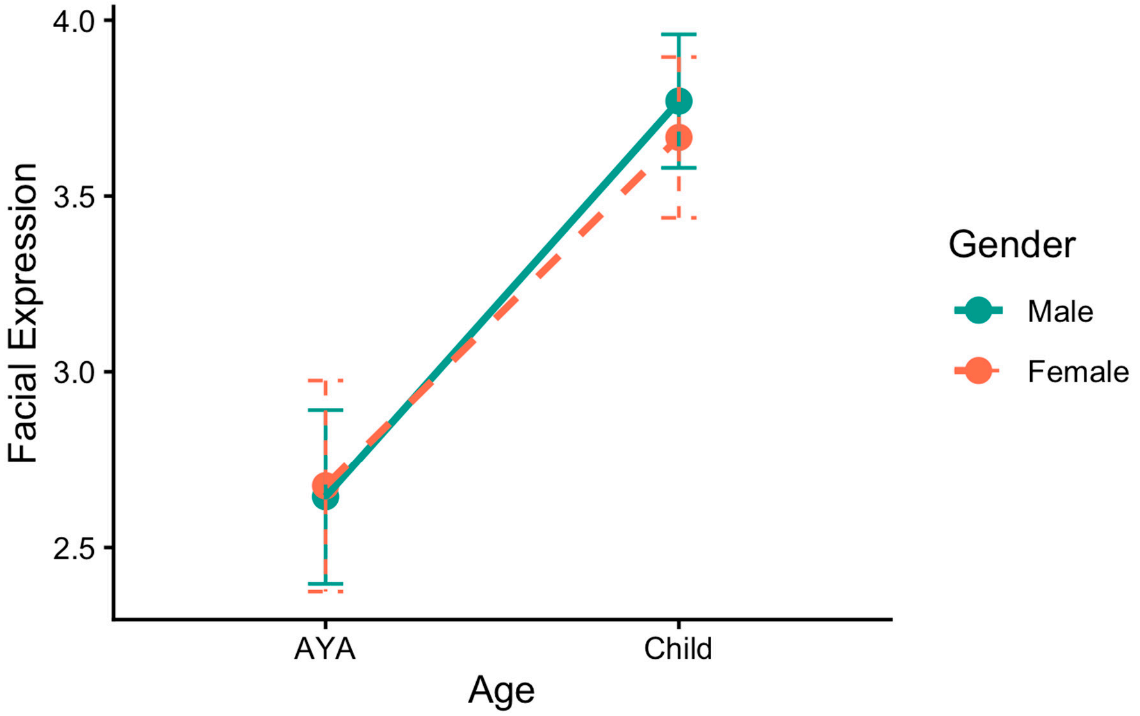

Figure S5. Estimated marginal means of Facial Expression according to age group and gender. The plot illustrates the interaction between age group and gender derived from the linear regression model, with points representing model-estimated mean scores and error bars indicating 95% confidence intervals. Solid and dashed lines represent males and females, respectively.

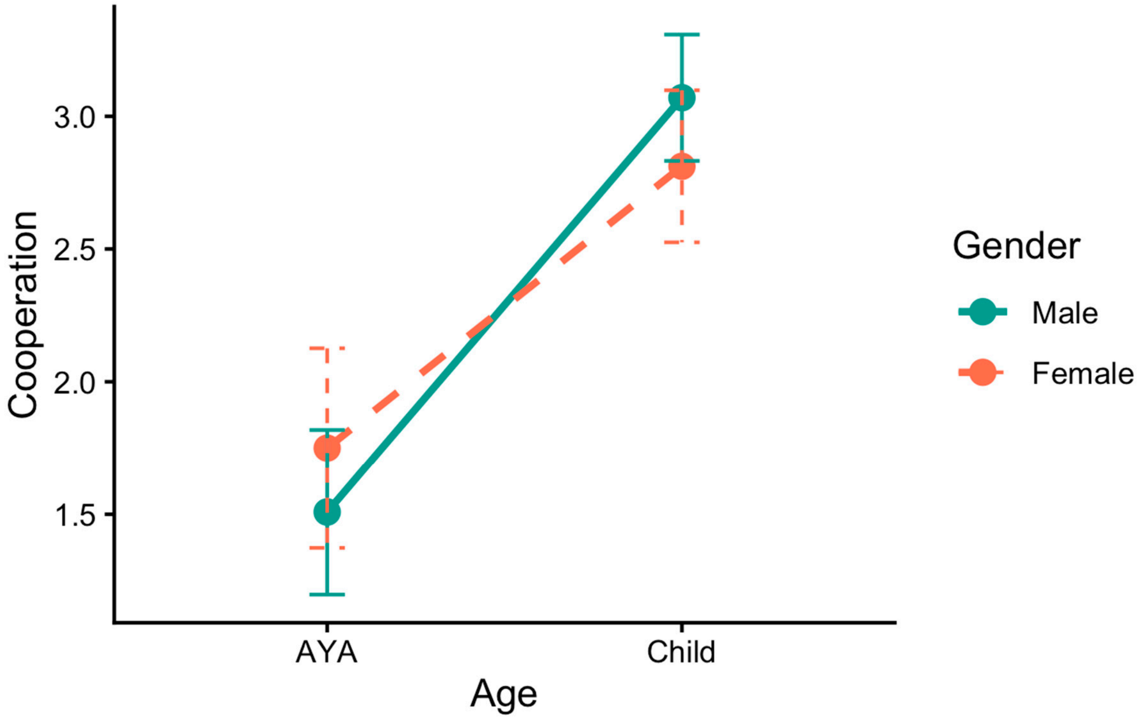

Figure S6. Estimated marginal means of Cooperation according to age group and gender. The plot illustrates the interaction between age group and gender derived from the linear regression model, with points representing model-

estimated mean scores and error bars indicating 95% confidence intervals. Solid and dashed lines represent males and females, respectively.

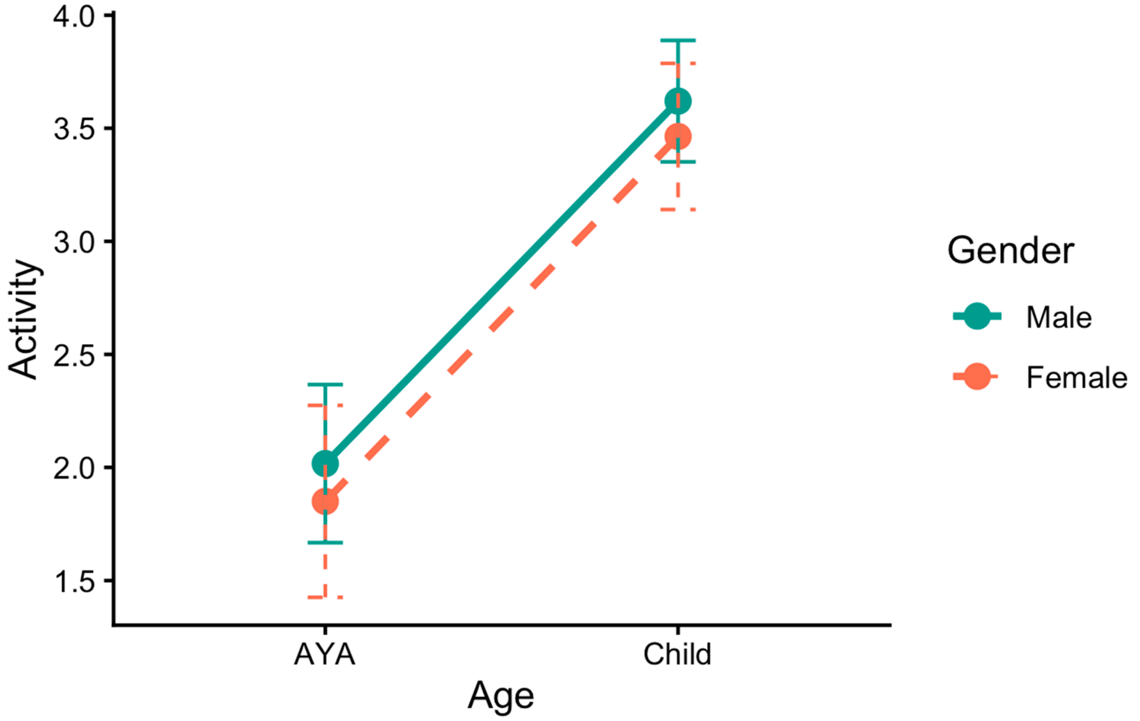

Figure S7. Estimated marginal means of Activity according to age group and gender. The plot illustrates the interaction between age group and gender derived from the linear regression model, with points representing model-estimated mean scores and error bars indicating 95% confidence intervals. Solid and dashed lines represent males and females, respectively.

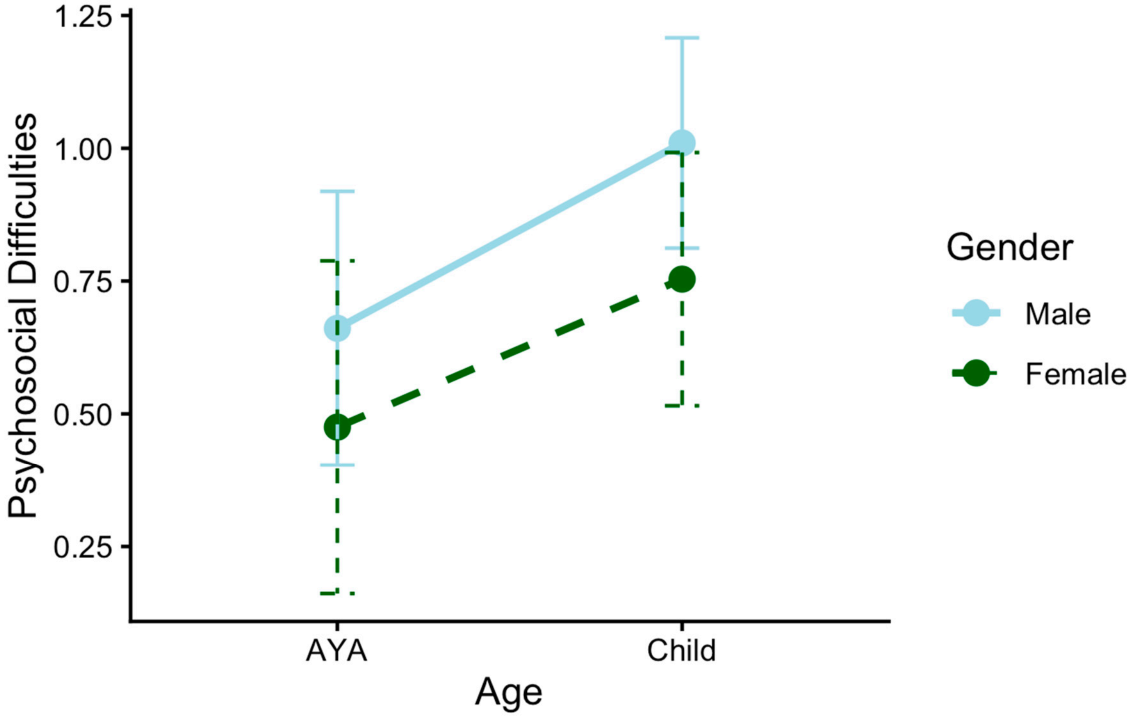

Figure S8. Estimated marginal means of Psychosocial Difficulties according to age group and gender. The plot illustrates the interaction between age group and gender derived from the linear regression model, with points representing model-estimated mean scores and error bars indicating 95% confidence intervals. Solid and dashed lines represent males and females, respectively.

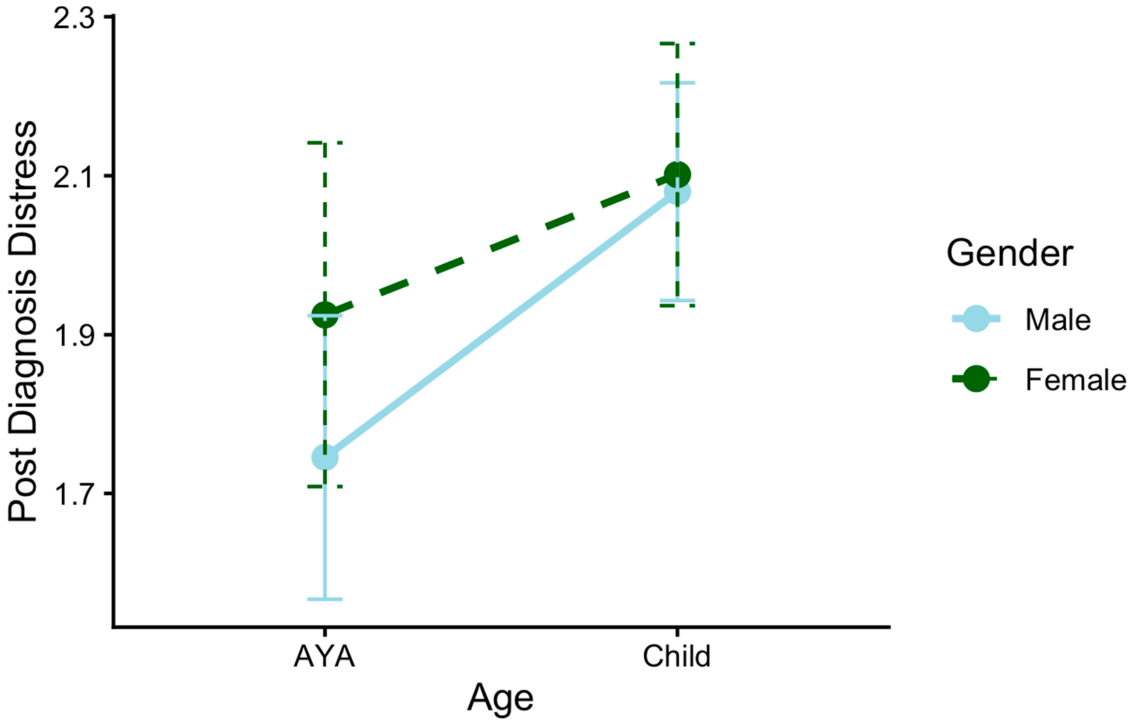

Figure S9. Estimated marginal means of Post Diagnosis Distress according to age group and gender. The plot illustrates the interaction between age group and gender derived from the linear regression model, with points representing model-estimated mean scores and error bars indicating 95% confidence intervals. Solid and dashed lines represent males and females, respectively.

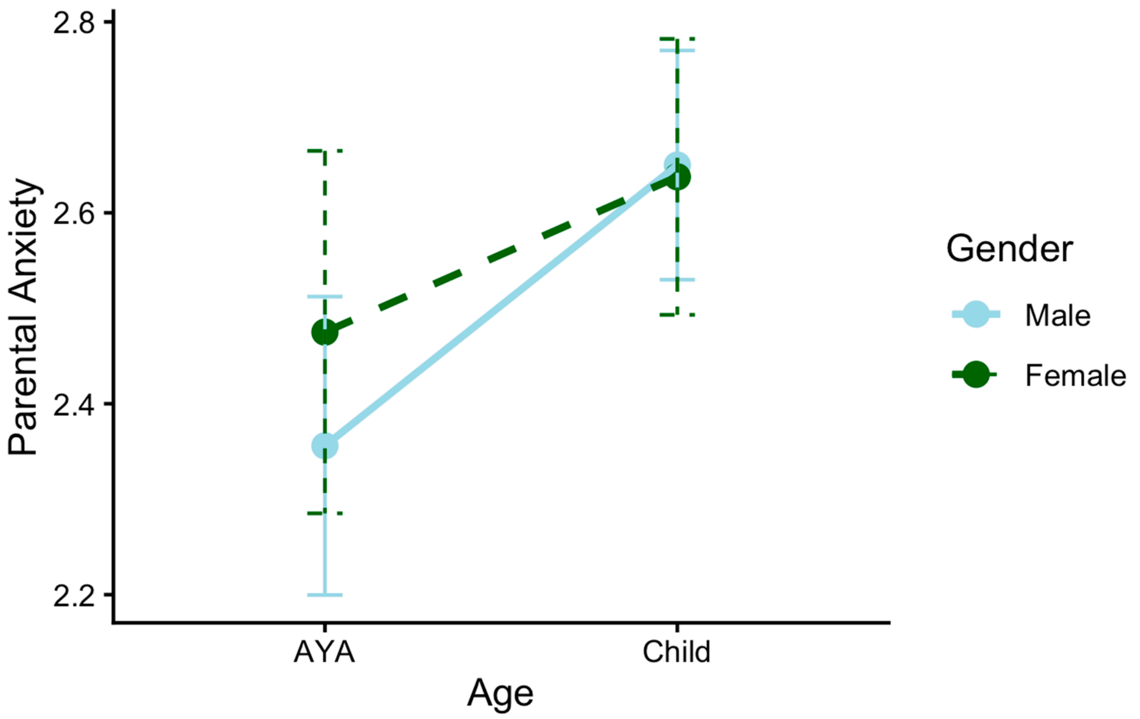

Figure S10. Estimated marginal means of Parental Anxiety according to age group and gender. The plot illustrates the interaction between age group and gender derived from the linear regression model, with points representing model-estimated mean scores and error bars indicating 95% confidence intervals. Solid and dashed lines represent males and females, respectively.

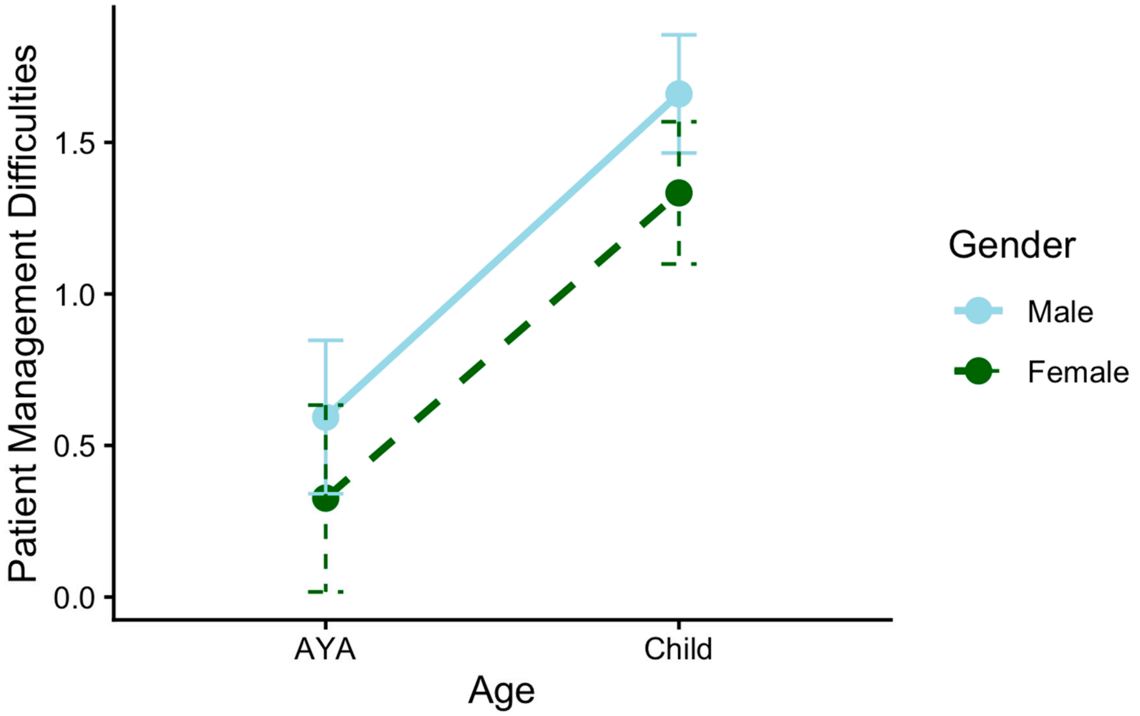

Figure S11. Estimated marginal means of Patient Management Difficulties according to age group and gender. The plot illustrates the interaction between age group and gender derived from the linear regression model, with points representing model-estimated mean scores and error bars indicating 95% confidence intervals. Solid and dashed lines represent males and females, respectively.

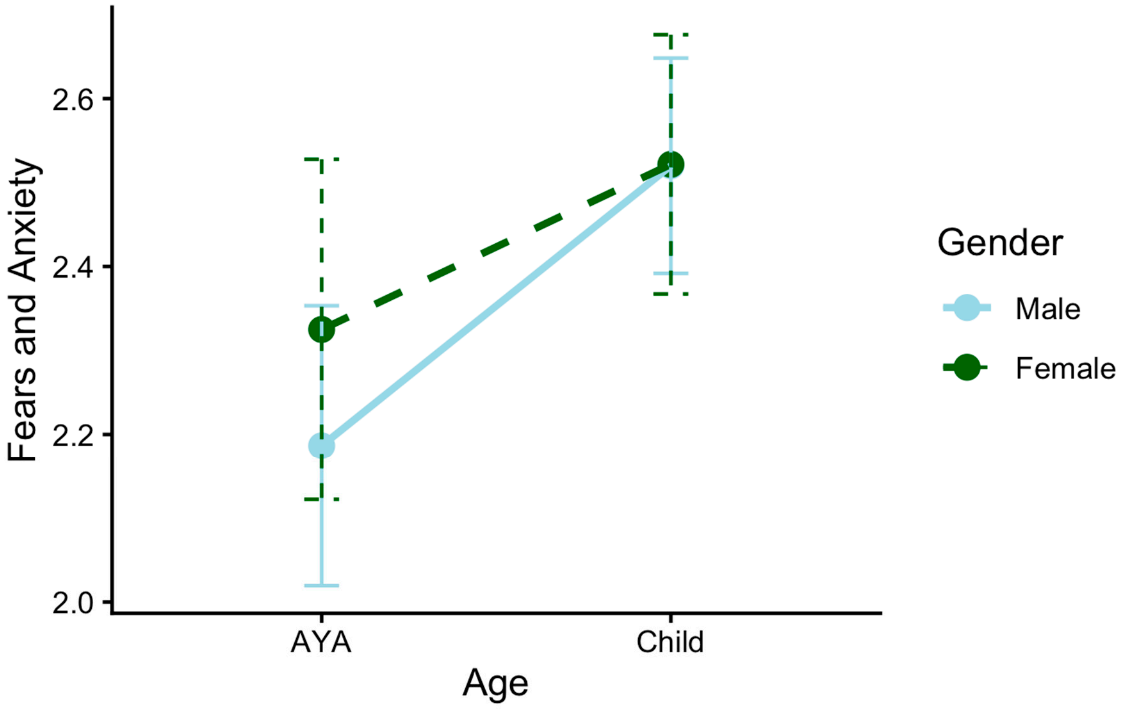

Figure S12. Estimated marginal means of Fears and Anxiety according to age group and gender. The plot illustrates the interaction between age group and gender derived from the linear regression model, with points representing model-estimated mean scores and error bars indicating 95% confidence intervals. Solid and dashed lines represent males and females, respectively.

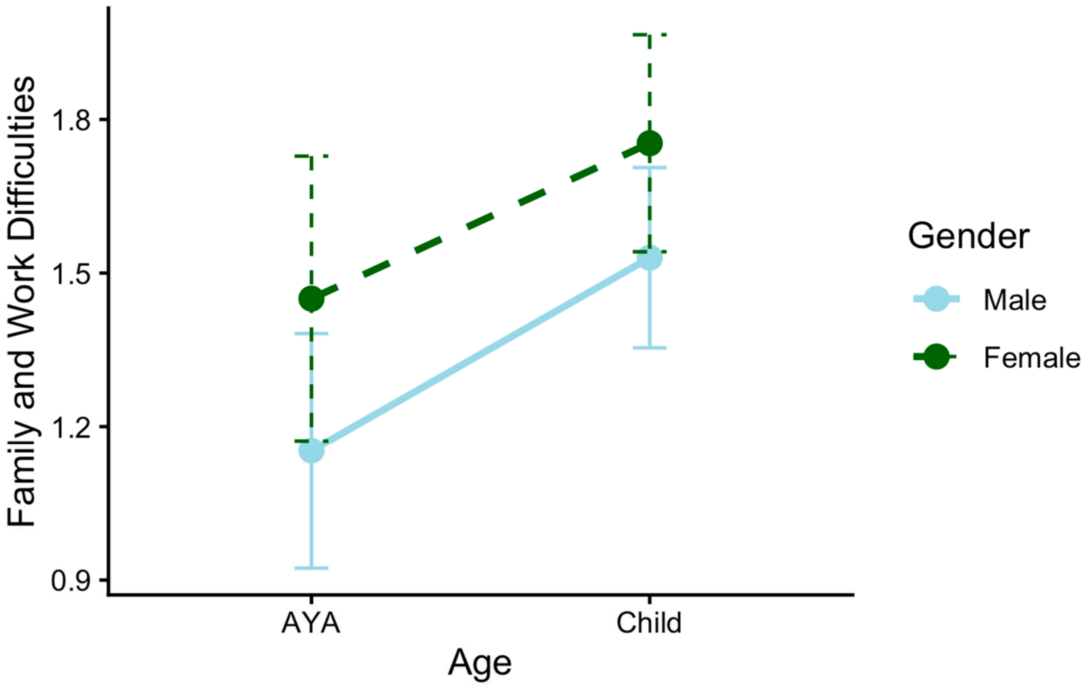

Figure S13. Estimated marginal means of Family and Work Difficulties according to age group and gender. The plot illustrates the interaction between age group and gender derived from the linear regression model, with points representing model-estimated mean scores and error bars indicating 95% confidence intervals. Solid and dashed lines represent males and females, respectively.

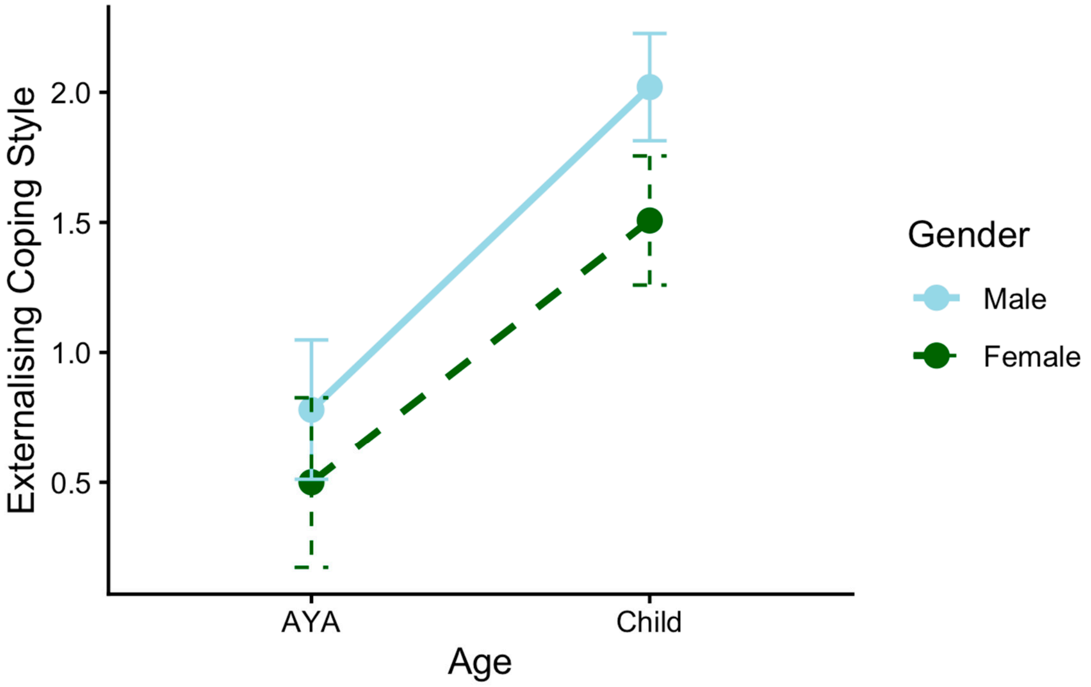

Figure S14. Estimated marginal means of Externalising Coping Style according to age group and gender. The plot illustrates the interaction between age group and gender derived from the linear regression model, with points representing

model-estimated mean scores and error bars indicating 95% confidence intervals. Solid and dashed lines represent males and females, respectively.

Table S1 reports the Benjamini–Hochberg correction applied to the age-group effect across all fifteen linear models, with unadjusted and adjusted p values side by side.

Table S1. Benjamini–Hochberg false-discovery-rate correction applied to the age-group effect across the 15 linear models. The family of tests comprises the ten items of the psychological interview (F1–F10) and the five CEMS subscales (E1–E5). The AYA group (≥12 years) is the reference category, so a positive β denotes a higher score in children ≤11 years.

**Outcome**	**β (children vs AYA)**	***p* (unadjusted)**	***p* (BH-adjusted)**	**R^2**
Psychological difficulties (F1)	0.35	0.036	0.044	0.036
Recent loss of autonomy (F2)	-0.05	0.764	0.764	0.012
Distress reactive to diagnosis (F3)	0.33	0.004	0.006	0.040
Externalising coping (F4)	1.24	< 0.001	< 0.001	0.246
Internalising coping (F5)	-0.19	0.169	0.195	0.018
Patient fear/anxiety (F6)	0.33	0.002	0.004	0.045
Parental fear/anxiety (F7)	0.29	0.004	0.006	0.039
Parenting difficulties (F8)	1.07	< 0.001	< 0.001	0.222
Family/social/work difficulties (F9)	0.38	0.011	0.015	0.053
Traumatic events before diagnosis (F10)	0.09	0.602	0.645	0.005
CEMS facial expression (E1)	1.13	< 0.001	< 0.001	0.227
CEMS vocalisation (E2)	1.56	< 0.001	< 0.001	0.236
CEMS activity (E3)	1.60	< 0.001	< 0.001	0.249
CEMS interaction (E4)	1.75	< 0.001	< 0.001	0.297
CEMS cooperation (E5)	1.66	< 0.001	< 0.001	0.272

R^2 values were derived from the model F statistics ($R^2 = kF / (kF + df_2)$, with $k = 3$ and $df_2 = 264$).
